# O‐GlcNAcylation: a bridge between glucose and cell differentiation

**DOI:** 10.1111/jcmm.12807

**Published:** 2016-03-01

**Authors:** Chao Sun, Jin Shang, Yuan Yao, Xiaohong Yin, Minghan Liu, Huan Liu, Yue Zhou

**Affiliations:** ^1^ Department of OrthopedicsXinqiao HospitalThird Military Medical UniversityChongqingChina; ^2^ Center for Evidence‐based and Translational MedicineZhongnan HospitalWuhan UniversityWuhanChina

**Keywords:** glucose, O‐GlcNAcylation, cell differentiation, osteogenic differentiation, chondrogenic differentiation, adipogenic differentiation

## Abstract

Glucose is the major energy supply and a critical metabolite for most cells and is especially important when cell is differentiating. High or low concentrations of glucose enhances or inhibits the osteogenic, chondrogenic and adipogenic differentiation of cell *via* the insulin, transforming growth factor‐β and peroxisome proliferator‐activated receptor γ pathways, among others. New evidence implicates the hexosamine biosynthetic pathway as a mediator of crosstalk between glucose flux, cellular signalling and epigenetic regulation of cell differentiation. Extracellular glucose flux alters intracellular O‐GlcNAcylation levels through the hexosamine biosynthetic pathway. Signalling molecules that are important for cell differentiation, including protein kinase C, extracellular signal‐regulated kinase, Runx2, CCAAT/enhancer‐binding proteins, are modified by O‐GlcNAcylation. Thus, O‐GlcNAcylation markedly alters cell fate during differentiation *via* the post‐transcriptional modification of proteins. Furthermore, O‐GlcNAcylation and phosphorylation show complex interactions during cell differentiation: they can either non‐competitively occupy different sites on a substrate or competitively occupy a single site or proximal sites. Therefore, the influence of glucose on cell differentiation *via* O‐GlcNAcylation offers a potential target for controlling tissue homoeostasis and regeneration in ageing and disease. Here, we review recent progress establishing an emerging relationship among glucose concentration, O‐GlcNAcylation levels and cell differentiation.

## Introduction

Glucose is a central source of energy and an important metabolite for all organisms. Other simple sugars and related molecules derived from sugars provide sources of energy for cells. Glucose also participates in the biosynthesis of polysaccharides, lipids, proteins and nucleic acids and the glucose concentration in the microenvironment, both *in vitro* and *in vivo*, markedly affects cell gene expression, proliferation, apoptosis and differentiation 1, 2, 3.

**Figure 1:**
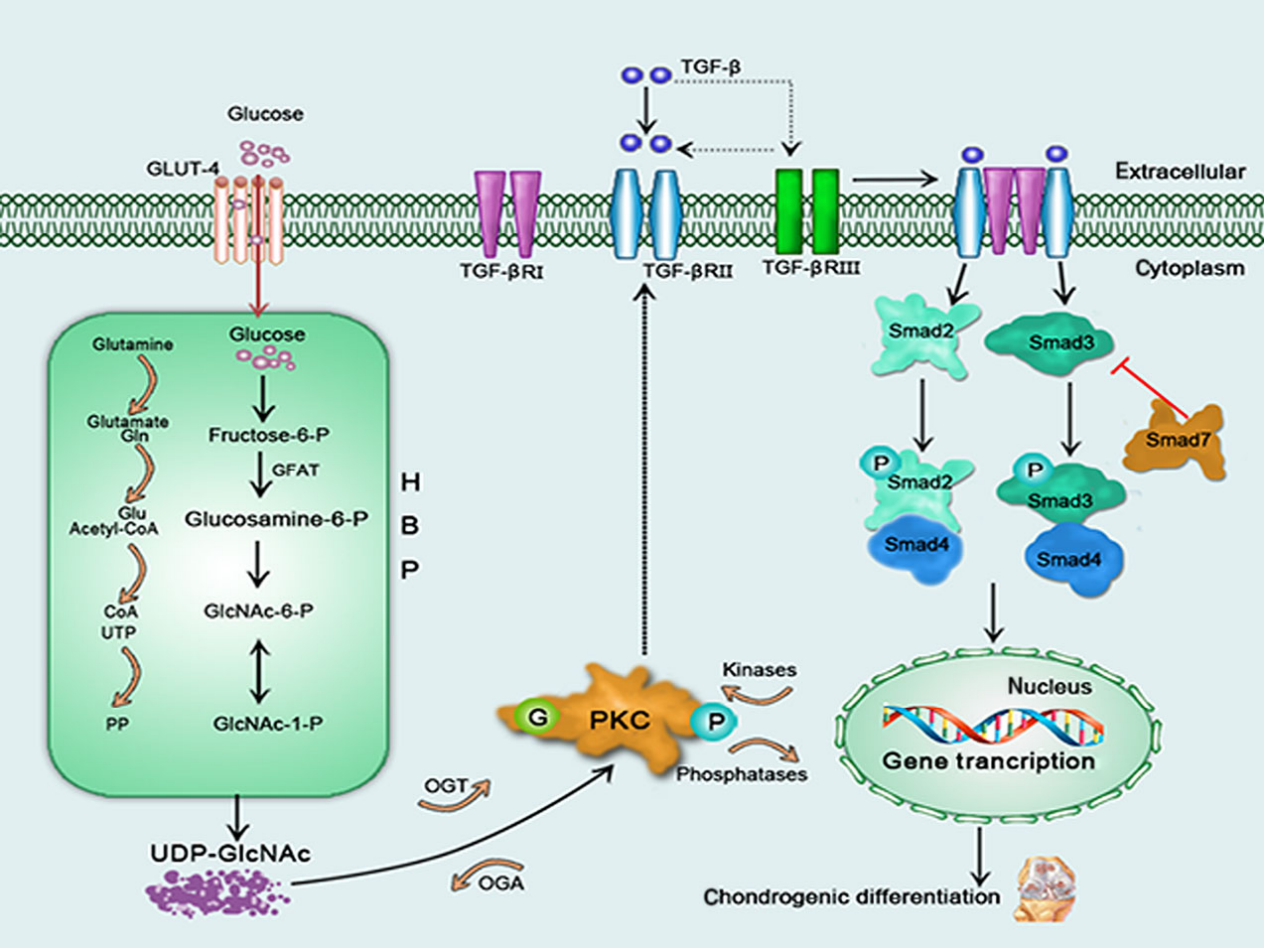
A schematic model illustrating O‐GlcNAc pathway and influence of glucose and O‐GlcNAcylation on chondrogenic differentiation. Glucose synthesizes UDP‐GlcNAc by HBP. O‐GlcNAc pathway consists of transfer and removal of O‐GlcNAc by OGT and OGA. Glucose decreases PKC activity with O‐GlcNAcylation by HBP, thus down‐regulating the expression of TGFβRII in cell pellets. The reduced TGFβRII expression results in decreased TGF‐β signalling upon the activation of TGF‐β ligand, further leading to reduced chondrogenesis.

Nuclear and cytoplasmic protein activities are dynamically regulated by the addition and removal of O‐linked‐β‐N‐acetylglucosamine (O‐GlcNAc) at serine and threonine residues 4 and the post‐transcriptional O‐GlcNAcylation of proteins markedly alters their function and fate. The enzymes responsible for this modification are O‐GlcNAc transferase (OGT) and O‐GlcNAcase (OGA), and the balanced O‐GlcNAc levels produced by their regulation are critical for metabolic homeostasis and other cellular processes. The addition and removal of O‐GlcNAc is sensitive to metabolic status 5, 6, altering the level of uridine 5′‐diphosphate‐GlcNAc (UDP‐GlcNAc) to activate O‐GlcNAcylation *via* the hexosamine biosynthetic pathway (HBP). In addition to the metabolic status, the removal of O‐GlcNAc is also regulated by the response of OGA to O‐GlcNAcylation levels (Fig. 1). O‐GlcNAcylation contributes to diverse intracellular functions *via* an assortment of targeted isoforms of enzymes in O‐GlcNAc and is critical to transcription, proliferation, differentiation and apoptosis 7. The O‐GlcNAc pathway regulates many important cellular pathways, including the insulin, transforming growth factor β (TGF‐β) and mitogen‐activated protein kinase (MAPK) signalling pathways. Lastly, O‐GlcNAcylation controls cells differentiation through the proteins and signalling pathways mentioned above in response to stress or changes nutrient levels.

The concentrations of glucose in common culture media range from 1.0 to 4.5 g/l (5.6–25 mM) 2. Specifically, based on conventional serum glucose levels, a glucose concentration of 5.5 mM is equal to approximately 0.99 g/l. Glucose concentrations of 11 mmol/l (1.98 g/l) or above are considered hyperglycaemic conditions. However, the higher end of the glucose concentration range (20–30 mM) is nearly equivalent to glucose levels of 3.6–5.4 g/l in clinical measurements. Regarding glucose concentrations related to cell culture medium, 5.5 mM is generally considered a low‐glucose culture medium, whereas 25 mM is considered a high‐glucose culture medium. The concentrations of glucose that is most often recommended and used for maintaining stem cell in culture is 5.5 mM, and is also called normal glucose 8.

During chondrogenic differentiation, cells migrate into the limb field and undergo a phenomenon termed ‘pre‐cartilaginous condensation’. The chondrocytes in the centre of the cartilaginous templates are stimulated to proliferate and then proceed through stages of maturation and hypertrophy. In the region of hypertrophy the chondrocytes are replaced by invading osteoblasts and the tissue is replaced by bone and bone marrow 9. And the stage of maturation of an osteocyte, which includes pre‐osteoblast proliferation, matrix formation and maturity and extracellular matrix mineralization from nascence until death, defines the morphology and function of the cell 10. In addition, the stage of adipogenic differentiation is composed of commitment pre‐adipocyte and terminal differentiation 11.

This review highlights the influence of glucose and O‐GlcNAcylation on cell differentiation, including osteogenic, chondrogenic and adipogenic differentiation.

## Chondrogenic differentiation

### Glucose concentration affects chondrogenic differentiation

The growth, development and structural integrity of joint are dramatically affected by the transport of glucose into chondrocytes and through articular cartilage 12, 13. In chondrogenic differentiation, glucose is the main precursor and a critical energy source for the synthesis of the extracellular cell matrix (ECM) and glycosaminoglycans 14, 15, 16. Thus, the concentration of glucose is essential for chondrocyte matrix synthesis, viability and differentiation. Studies have demonstrated that high concentrations of glucose reduce the chondrogenic potential of human mesenchymal stem cells (MSCs) 17, muscle‐derived stem cells 18, and adipose tissue‐derived MSCs (ASCs) 19. And, low concentrations of glucose have been reported to increase the chondrogenic potential of MSCs 20.

Studies *in vitro* and vivo have linked hyperglycaemia with local and systemic toxicities relevant to OA, caused by high‐glucose concentration 21. Hyperglycaemia decreases transport of dehydroascorbate into chondrocytes, compromising the synthesis of type II collagen and increasing levels of reactive oxygen species (ROS) and inflammatory mediators to mediate cartilage destruction 22, 23. Insulin‐like growth factor‐1 (IGF‐1) and insulin play an important role in chondrogenic differentiation. Insulin‐like growth factor‐1 stimulates the chondrogenic differentiation of MSC into chondrocytes pre‐hypertrophic and hypertrophic chondrocytes by stimulating proliferation, regulating cell apoptosis, inducing expression of chondrocyte markers and enhancing extracellular matrix biosynthesis 24, 25. Insulin is structurally similar to IGF‐1 and can activate the IGF‐1 receptor, and insulin has been shown to be an essential additive for chondrogenic differentiation of mesenchymal progenitor cells and that it influences the grade of chondrogenic differentiation dose‐dependently 26. Previous studies have shown that there is an accumulation of O‐GlcNAcylated proteins in the cartilage of human osteoarthritic patients 27. It has been reported that the expression and activity of matrix metalloprotease (MMP) 2 and MMP9 28 and the progression of chondrogenic differentiation 29 are enhanced by OGA inhibition, which could increase the intracellular level of O‐GlcNAcylation. In addition, it has been reported that insulin and thiamet‐G (an inhibitor of OGA) produce a obvious difference in the activation proteoglycan synthesis although little difference in the extent of differentiation markers inductions in ATDC5 cells. Then, the mechanisms by which glucose and O‐GlcNAcylation influences chondrogenic differentiation are discussed below.

Activation of TGF‐β signalling pathway is critical for chondrogenic differentiation of MSCs 30. High‐glucose culture induces hypertrophy of mouse embryonic fibroblasts and rat kidney epithelial cells by up‐regulating TGF‐β signalling pathway 31. High‐glucose culture also modulates PKC activity to up‐regulate the expression of TGF‐β receptor expression of vascular smooth muscle cells 32. Mesenchymal stem cells cultured in high glucose prior to differentiation show decreased chondrogenesis 19. High‐glucose expansion culture reduces PKC activity to chondrogenic induction, resulting in down‐regulating the expression of TGFβRII in MSCs. Then TGF‐β signalling upon the activation of TGF‐β ligand was decreased by the reduced TGFβRII, further leading to reduced chondrogenesis 17 (Fig. 1).

**Figure 2:**
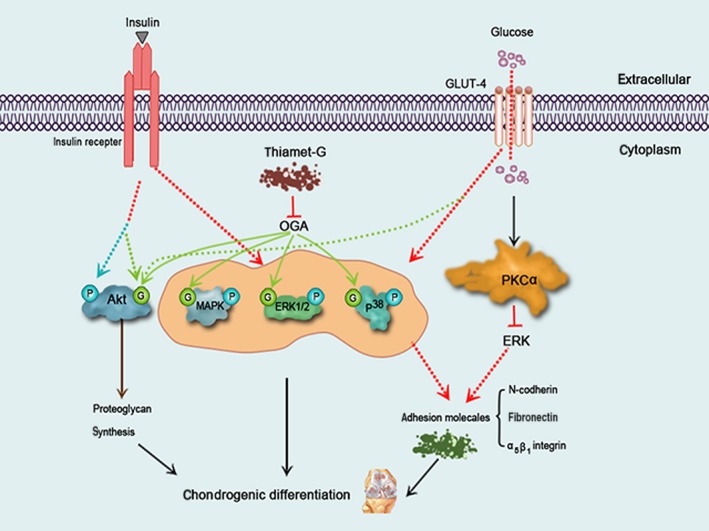
A schematic model illustrating the effect of glucose, thiamet‐G and insulin on O‐GlcNAcylation and phosphorylation of signalling molecules during chondrogenesis. High glucose up‐regulates p38 and down‐regulates ERK activity through PKCα, priming stimulating chondrogenesis by increasing the expression of adhesion molecules. Insulin and glucose/thiamet‐G stimulate chondrogenic differentiation by inducing O‐GlcNAcylation and phosphorylation and of signalling molecules, including MAPK, p38 and ERK1/2. Insulin induces O‐GlcNAcylation and phosphorylation of Akt, while high glucose and thiamet‐G simply induce Akt O‐GlcNAcylation. Then activated‐Akt stimulates proteoglycan synthesis in chondrocytes.

However, in another report, high concentrations of glucose was shown to enhance chondrogenesis in chick mesenchymal cells. High glucose has been shown to up‐regulate p38 and down‐regulate extracellular signal‐regulated kinase (ERK) activity through PKCα, priming the stimulation of chondrogenic differentiation by modulating the expression of adhesion molecules 33 (Fig. 2). In addition, chondrogenesis might be modulated by complex protein kinase signalling cascades, including those downstream of ERK 34, PKC 35 and p38 36. The expression levels of cell adhesion molecules, including fibronectin, N‐cadherin and α5β1 integrin are positively regulated by PKC in mesenchymal cells 34, 35, 36. Extracellular signal regulated kinase negatively modulates chondrogenesis by altering the expression of cell adhesion molecules, whereas p38 plays an opposite role at the post–pre‐cartilage condensation stage 36 (Fig. 2). Activation of p38 is necessary for the accumulation of sulphated proteoglycans and cellular condensation. In addition, long‐term effect of high‐glucose concentration on human media artery smooth muscle cells down‐regulates of basal RAC‐α serine/threonine‐protein kinase (Akt) phosphorylation, while acute stimulation of cells in high glucose with insulin‐activated Akt 37. The different effect of high glucose on MSCs and chick mesenchymal cells may be caused by different cell and induction (TGF‐β/insulin). And it remains to be further exploring.

At last, high concentration glucose can also increase the formation of advanced glycation end‐products (AGEs) in diabetes or *in vitro* models 38. It is reported that the proteoglycan synthesis and degradation of articular cartilage were negatively affected by an increase in AGE levels in OA patients 39. And chondrogenic differentiation in AGE‐2–treated or AGE‐3–treated MSCs were inhibited 40.

### O‐GlcNAcylation affects chondrogenic differentiation

Early hypertrophic chondrocytes accumulate glycogen occurs during the maturation phase, and it seems plausible that proteins can be O‐GlcNAcylated during chondrogenic differentiation 12. Recent findings demonstrate MAPK, ERK1/2 and p38 could be O‐GlcNAcylated 41, 42. Insulin and thiamet‐G can induce increases in p‐MAPK, p‐ERK1/2 and p‐p38 in some cell types 43, 44. However, thiamet‐G and other OGA inhibitors failed to induce Akt phosphorylation 45; intriguingly, some studies have shown that Akt phosphorylation is critical for insulin‐induced proteoglycan synthesis in chondrocytes 46 (Fig. 2).

Previous studies show there is an extensive cross‐talk between O‐GlcNAcylation and the phosphorylation of Akt, with both modulating its function 47, 48, and it has been proposed that Akt O‐GlcNAcylation and phosphorylation can be simultaneously induced 45. Same site competition, proximal site competition and proximal site occupation are interrelationships between O‐GlcNAcylation and phosphorylation. The balance between O‐GlcNAcylation and phosphorylation can change the cellular function of the protein 49. O‐GlcNAcylation of Akt has no effect or a stimulating effect on its enzymatic activity but did not inhibits its phosphorylation 48. Furthermore, Akt O‐GlcNAcylation was even more intense when Akt phosphorylation was activated in insulin‐induced chondrogenic differentiation. However, some reports have shown that decreases in Akt phosphorylation and/or Akt activity is correlation with an increase in Akt O‐GlcNAcylation 50, 51, 52 (Fig. 2). O‐GlcNAcylation not only modulates Akt activity but also modulates the cellular distribution of the enzyme. Such processes may induce further changes in the targets of Akt 45. PKC also plays key regulatory roles in major signal transduction pathways controlling a wide range of biological responses including gene expression, cell morphology, proliferation and differentiation 53. It is reported that all PKC isozymes are dynamically modified by O‐GlcNAc, and O‐GlcNAc modifications correlate negatively with PKCα activity in rat hepatocytes 54.

## Osteogenic differentiation

### Glucose concentration affects osteogenic differentiation

Bone is affected by diabetes in both humans and animal models, leadings to osteoporosis and osteopaenia 55, 56, 57, 58. Diabetes alters biochemical markers 59 and mineral density of bone in humans, and the poor glycaemic control in diabetes mellitus contributes to reduced bone mass and frequently to fractures. We therefore attribute this complication to the high blood glucose concentrations in diabetic patients. Indeed, glucose is reported to have a direct activating effect on osteoclasts and acts as a principal energy source for osteoclastic bone resorption 60. It is reported that glucose inhibits collagen fibril formation and subsequent cross‐linking in human osteoblast‐like cells *in vitro* 61. Furthermore, the osteoblastic cell proliferation‐induced production of IGF‐1 and the basal and osteocalcin secretion‐induced production of 1,25(OH)2D are inhibited in human MG‐63 cells in a high‐glucose environment *in vitro* 62, 63.

In recent decades, scientists have paid increasing attention to the influence of glucose on cells. It has been reported that the proliferation and differentiation of MSCs, which are the common starting point in the development of osteoblasts, are down‐regulated in the streptozotocin‐induced diabetic mouse 30, 64, 65. Furthermore, high‐glucose concentrations reduce the osteogenic potential of human MSCs 20, mouse bone marrow‐derived MSCs 66, 67, 68 and ASCs 19, along with subsequent diminished mineralization. In addition, low‐glucose media leads to a higher degree of differentiation by human bone marrow MSCs and mouse MSCs compared with osteocytes in normal‐ and high‐glucose media 69, 70. Another report demonstrated that glucose restriction increases the osteogenic capacity of mouse MSCs *in vitro*.

High concentrations of glucose alter the differentiation of MSCs into osteoblast lineages and their mineralization into nodules. High glucose also interferes with the formation and mineralization of the extracellular matrix. The deleterious effect of high glucose on BMSC‐derived osteoblast proliferation and function can be ameliorated by insulin 66, which controls blood glucose levels and maintains the levels of vitamin 1, 25(OH)2D, IGF‐1, and parathyroid hormone (PTH) to indirectly regulate bone development and formation in patients and rats *in vivo* 71, 72, 73. Furthermore, insulin treatment of human and mouse osteoblasts down‐regulates apoptosis, increases the presence of transporter molecules, induces the synthesis of collagen and insulin‐like growth factor‐binding protein‐3 (IGFBP‐3), increases proliferation and sensitizes cells to PTH 74, 75, 76, 77, 78, 79. Finally, glucose regulates the distribution pattern of insulin receptors in MSCs during osteogenic differentiation.

Runt‐related transcription factor‐2 (Runx2) is a member of the runt‐domain gene family of DNA‐binding proteins (Runx1, Runx2, Runx3), which control the expression of numerous genes involved in cell growth, proliferation and determination of cell lineage 80. OSE2 is the specific DNA‐binding site for Runx2 81. It is reported that high‐glucose (11 mmol/l) stimulates Runx2 expression, while higher glucose (44 mmol/l) inhibits Runx2 expression 82. And high glucose can also enhance phosphorylation of CREB 83. Long‐term incubation of human and mouse osteoblasts with AGEs decreases cellular activity, proliferation, the expression of collagen type I, osteocalcin and IGF‐1, alkaline phosphatase (ALP) activity, and the formation and mineralization of the ECM 84, 85, 86. Advanced glycation end‐products increase ALP activity and intracellular calcium content, while decrease mineralization and mature bone nodule formation in MSCs differentiation 40.

Hyun‐Jung *et al*. used two‐dimensional electrophoresis for a proteomic analysis of proteins in MSCs affected by calorie restriction 70 and found seven proteins to be down‐regulated: laminin‐binding protein 87, mutant beta‐actin 88, Sec‐12 protein 89, alpha soluble N‐ethylmaleimide‐sensitive fusion protein (SNAP) 90, manganese superoxide dismutase (MnSOD) 91, proteasome alpha 1 subunit 92 and ribosomal protein S12 93. These authors also observed the up‐regulation of three other proteins: aldehyde dehydrogenase (ALDH) 94 and the prolyl 4‐hydroxylase alpha (P4HA) 95 subunit, under normal‐glucose and low‐glucose conditions (Table 1). These proteins are critical for cell division, development, differentiation, protein synthesis, protein folding and assembly and the stress response. The potential of MSCs to differentiate into osteocytes may be influenced by differentially expressed proteins under low concentrations of glucose.

**Table 1:** Expression of proteins in MSCs under normal glucose (5.5 mM) and low glucose (1.4 mM) conditions during osteogenic differentiation

Protein	MW (kD)	Characteristic	Regulation	Reference
Aldehyde dehydrogenase	57.6	Protecting or detoxifying enzyme; preserves stem cells from cytotoxic effects	Up	70
Prolyl 4‐hydroxylase alpha subunit	61.1	Intracellular enzyme; required for synthesis and formation of all known types of collagen	Up	71
Laminin binding protein	31.9	Extracellular protein; affects cell‐substratum attachment, spreading, migration, differentiation, proliferation, and neurite outgrowth	Down	63
Mutant beta‐actin	42.1	Cytoskeletal protein; participates in muscle contraction, cell motility, cytokinesis, vesicle and organelle movement, cell signalling, establishment and maintenance of cell junctions and cell shape	Down	64
Sec 12 protein	80	Guanine nucleotide exchange factor; promotes the recruitment of COPII vesicle coats and cargo selection	Down	65
Alpha soluble N‐ethylmaleimide sensitive fusion protein	33.7	Homohexameric AAA ATPase; a central component of the cellular machinery in the transfer of membrane vesicles from one membrane compartment to another	Down	66
Manganese superoxide dismutase	24.9	Vesicle coats and cargo selection	Down	67
Proteasome alpha 1 subunit	29.8	Intracellular protien; modifies proteasome	Down	68
Ribosomal protein S12	14.9	Locates in the cytoplasm; belongs to the S12E family of ribosomal proteins	Down	69

### O‐GlcNAcylation affects osteogenic differentiation

An increasing number of studies report that the skeleton can act as a nutrient stress sensor that associates bone metabolism, bone mineral homeostasis and whole‐body nutrient status through bone‐specific endocrine signals or other signalling pathways 96, 97, 98, 99, 100. Among them, the role of protein glycosylation in osteoblast function may indicate that whole‐body glucose homeostasis can affects bone metabolism 96, 97, 99, 101, 102.

It has been proposed that dynamic O‐GlcNAcylation is sensitive to nutrient status, including extracellular glucose flux, *via* the HBP 103. O‐GlcNAcylation may act as a nutrient‐responsive regulatory mechanism in the skeleton because insulin receptor substrates are O‐GlcNAcylated 104, and insulin receptor substrates are critical mediators of insulin/IGF‐1 signalling. It has also been reported that many proteins are O‐GlcNAcylated in osteoblasts and that the extent of protein O‐GlcNAcylation varies during osteoblast differentiation 105. O‐GlcNAcylation of protein may induce osteocalcin. It is evidently based on an observed increase in global protein O‐GlcNAc modification, including CREB and TAK1 signalling complex, in osteoblasts cultured in high concentrations of glucose compared to low concentrations of glucose 106. Thus, O‐GlcNAcylation may offer a potential target for controlling bone development at the osteoblast level.

**Figure 3:**
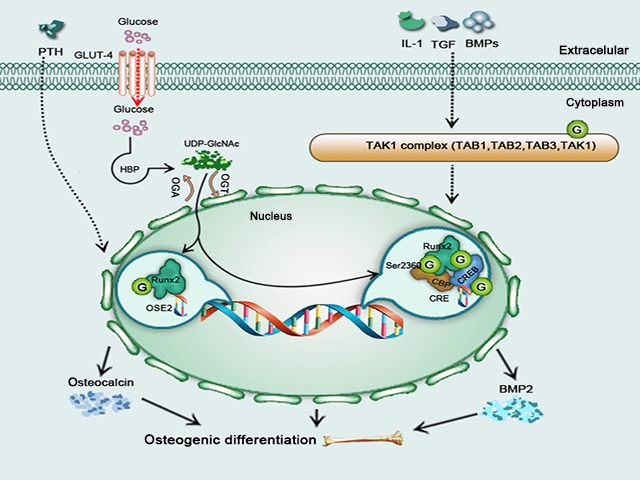
A schematic model illustrating influence of O‐GlcNAcylation on osteogenic differentiation. Elevated O‐GlcNAc increases osteocalcin transcription *via* OSE2 and Runx2. IL‐1, TGF and BMPs influence the O‐GlcNAcylation of Runx2, CBP and CREB *via* the TAK1 complex to increase BMP2 transcription, with all enhancing osteogenic differentiation.

It has been reported that the transcriptional activity of Runx2 is enhanced in osteoblast differentiation *via* PTH stimulation with an OGA inhibitor 105. Furthermore, previous studies have demonstrated that elevated O‐GlcNAcylation of proteins enhances the expression of differentiation markers in pre‐osteoblasts and have suggested that O‐GlcNAcylation of Runx2 and osteoblast‐specific cis‐element 2 (OSE2) contributes to osteoblast differentiation. OSE2 region of the osteocalcin promoter is important for elevated O‐GlcNAcylation, priming inducing osteocalcin 107. Runx2 transcriptional activity is modified by elevated O‐GlcNAcylation, and the transcription of osteoblast‐specific markers (such as osteocalcin) can be stimulated by the binding of Runx2 to specific enhancer regions of the gene (OSE2). Thus, the transcription of osteocalcin is increased by elevated O‐GlcNAcylation and mediated by Runx2 and OSE2 81, 108 (Fig. 3).

It has been proposed that osteoblast function is regulated by the O‐GlcNAcylation of TGF‐β‐activated kinase‐1/MAP3K7‐binding protein‐1 and ‐2 (TAB 1/TAB 2), which are associated with the TGF‐β‐activated kinase 1 (TAK1) signalling node 109. It is intriguing that TAK1 interferes with osteoblast differentiation by regulating Runx2 activation and its association with the cAMP response element‐binding protein (CREB)‐binding protein (CBP) 110, a transcriptional co‐activator and histone acetyltransferase, plays a crucial role in osteoblast differentiation. CBP is O‐GlcNAcylated at its C‐terminal domain, at Ser‐2360, which is also a phosphorylation site 111, 112. O‐GlcNAcylation and phosphorylation thus may interact at Ser‐2360 to affects CBP function. Osteoblast differentiation, mineralization and skeletal development are influenced by TAK1‐modulated transcription by enhancing the association between Runx2 and CBP 110. Recent findings demonstrate that TAB 1 113, 114, 115, TAB 2 109, TAB 3 113, 114 and TAK1 113 are O‐GlcNAcylated and that TAB 2 is essential for osteogenic differentiation 114. IL‐1, TGF and BMPs stimulate the TAK1 complex (Fig. 3).

It has been proposed that CBP interacts with and regulates the transcriptional activity of Runx2 and CREB and that it also enhances CREB‐mediated BMP2 116. Post‐translational modifications modulates (PTMs) the activity and protein interactions of CBP; as one class of PTM, O‐GlcNAcylation modifies CBP‐, CREB and CREB‐regulated transcription coactivator‐2 (CRTC2) 117 and OGT has also been shown to co‐localize with CREB at unique promoter regions 118. Although the O‐GlcNAcylation of signalling regulator such as CBP is regarded as a mechanism controlling the fate of osteoblast, CBP is also affected by PTH 119, insulin/IGF‐1 116, BMPs 110 and Wnts 120 (Fig. 3).

## Adipogenic differentiation

### Glucose concentration affects adipogenic differentiation

Increased adipose accumulation in marrow has recently been shown in a streptozotocin‐induced insulin‐dependent diabetes mellitus mouse model 121. As a high level of glucose in the blood is a major characteristic of diabetes, the glucose concentration may have an important influence on adipogenic differentiation. It has been reported that in comparison to a low‐glucose culture medium, a high‐glucose medium enhances the adipogenesis of mouse muscle‐derived stem cells, mouse bone marrow‐derived MSCs 122 and human ASCs 18, 19. And, in another report adipogenic capacity was impaired by transfer to a low‐glucose medium 20.

**Figure 4:**
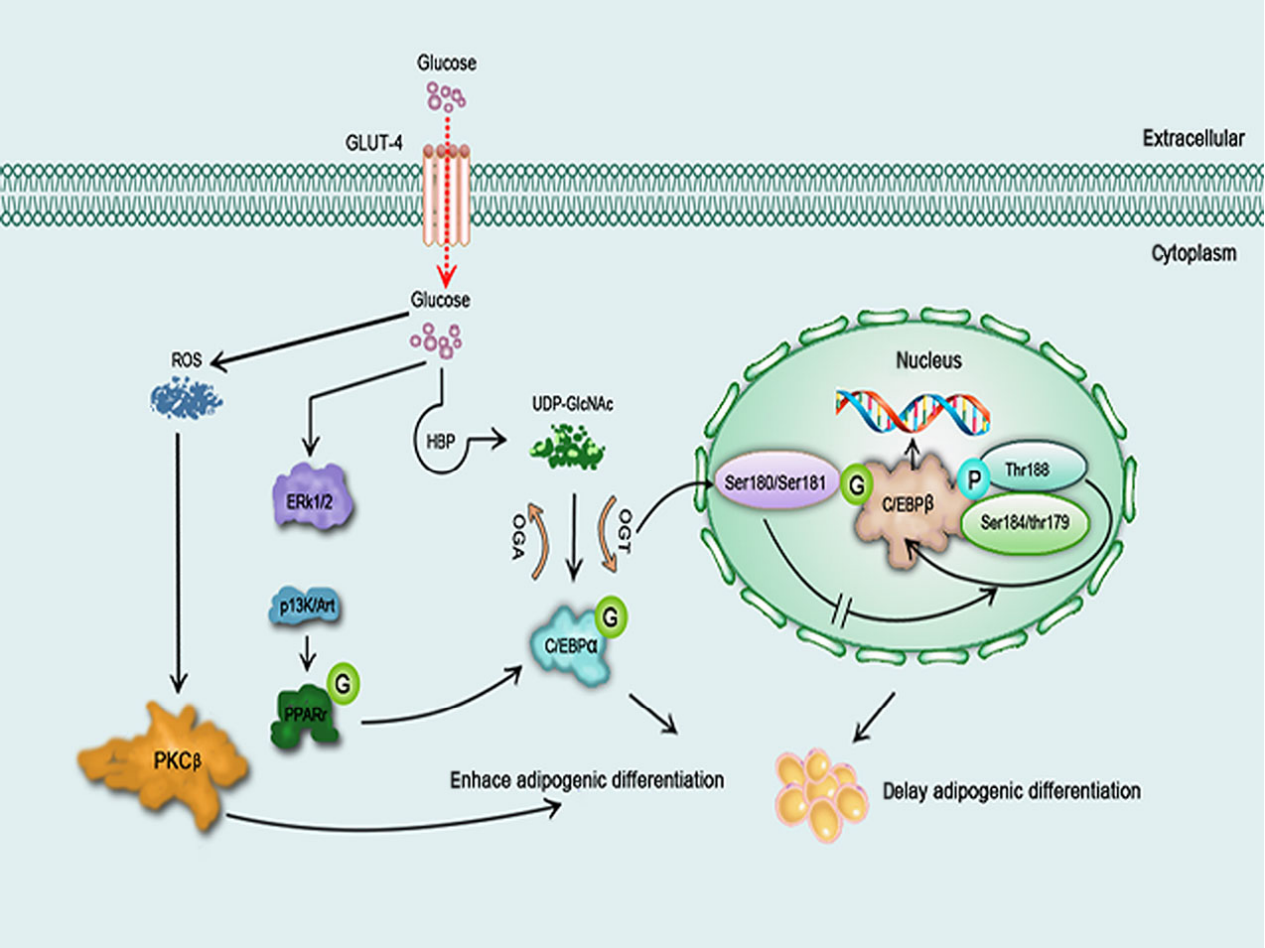
A schematic model illustrating influence of glucose and O‐GlcNAcylation on adipogenic differentiation. High concentrations of glucose enhances adipogenic differentiation through the ERK‐mediated PI3K/Akt pathway or the ROS/PKCβ pathway. O‐GlcNAcylation of C/EBPα promotes adipogenic differentiation, but O‐GlcNAcylation of C/EBPβ inhibits autophosphorylation thereby delaying adipogenic differentiation.

PKC activation and ROS production are crucial steps in adipogenesis, and both processes are induced by high glucose. The neoformation of adipose cells is enhanced by ROS *via* downstream signalling molecules particularly PKCβ 18, and previous studies have demonstrated that PKC plays a critical role in adipogenic differentiation and diabetes. Additionally, there are close relationship among ROS production, PKC and adipogenesis 65 (Fig. 4). Peroxisome proliferator‐activated receptor (PPAR) and CCAAT/enhancer‐binding proteins (C/EBPs) are also crucial for adipogenic differentiation 123, 124, 125. A recently study demonstrates that the mRNA and protein levels of C/EBPs and PPARγ were increased during adipocyte differentiation 126. C/EBPα is key to the production of specific adipogenic genes, and its expression is induced by PPARγ, which is regulated by MEK/ERK signalling pathway and by C/EBPβ during adipogenic differentiation 127, in late‐stage adipogenesis. The ERK signalling pathway has both positive and negative functions in the adipocytic differentiation of MSCs. Adipocyte differentiation is regulated at each step by the MAPK signalling pathway 128. Furthermore, activation of insulin receptor substrate‐1 (IRS‐1)/phosphatidylinositol 3‐hydroxy kinase (PI3K)/Akt plays a crucial role in lipid synthesis stimulated by insulin 129. The expression of the fork‐head transcription factor gene Foxc2 is induced by tumour necrosis factor‐α (TNF‐α) and insulin *via* PI3K/ERK1/2 signalling pathways in 3T3‐L1 adipocytes 130. Therefore, high concentrations of glucose enhance the accumulation of lipid in adipogenesis *via* an ERK1/2‐activated PI3K/Akt‐regulated PPARγ signalling pathway in mouse bone marrow‐derived MSCs 122 (Fig. 4).

### O‐GlcNAcylation affects adipogenic differentiation

Glucose uptake, lipid storage and insulin sensitivity are affected by the activation of HBP *via* the administration of glucosamine or the overexpression of glutamine 6 fructose phosphate transaminase 1 (GFAT‐1) in adipocytes 131, 132, 133, 134, and the O‐GlcNAcylation of proteins may be intimately connected to this phenomenon. Indeed, recent findings demonstrate that O‐GlcNAc‐modified proteins are modulated throughout development in a complex pattern. Aberrant O‐GlcNAcylation may affect cell differentiation, which may lead to developmental abnormalities 135. It has been observed that protein O‐GlcNAcylation dynamically increases when 3T3‐L1 pre‐adipocytes are induced to differentiate, and O‐GlcNAcylation of protein may play an important role in adipocyte differentiation with this elevation persisting for the entire differentiation period 126. Furthermore, the formation of lipids in adipocytes is prevented by GFAT‐1 siRNA and GFAT‐1 inhibitors although a reduction in protein O‐GlcNAcylation. The expression of C/EBPβ and PPARγ was reduced by GFAT‐1 siRNA treatment in adipocytes, suggesting that the HBP may regulate adipocyte differentiation partly by altering the expression of C/EBPβ and PPARγ. Such findings shows that the timing of the increase in O‐GlcNAcylation is associated with the timing of C/EBPα expression in adipogenesis and that an inhibitor of GFAT‐1 can block the O‐GlcNAcylation‐induced adipocyte differentiation. Thus, O‐GlcNAcylation may play an important role in adipogenic differentiation by affecting C/EBPα expression 136.

Recently, it has been proposed that C/EBPβ O‐GlcNAcylation delays adipocyte differentiation 137. C/EBPβ is sequentially phosphorylated on Thr188/Ser184/Thr179; and C/EBPβThr188 phosphorylation primes phosphorylations on Ser184/Thr179. Phosphorylations on Thr188/Ser184/Thr179 of C/EBPβ are key to the binding activity between C/EBPβ and DNA. C/EBPβ is itself O‐GlcNAcylated at Ser180 and Ser181, and the phosphorylation and O‐GlcNAcylation sites are very close, both being located in the regulatory domain. O‐GlcNAcylation of C/EBPβ inhibits the phosphorylations of itself, but it does not affect its DNA‐binding activity. Elevated O‐GlcNAcylation of C/EBPβ markedly reduces both the phosphorylation and DNA‐binding activity of itself. As a result, elevated C/EBPβ O‐GlcNAcylation delays the adipocyte differentiation programme. Furthermore, mutations on Ser180 and Ser181 significantly enhance the transactivation activity of C/EBPβ, indicating that the blockade O‐GlcNAcylation promotes this phosphorylation. In conclusion, O‐GlcNAcylation and phosphorylation compete for occupation of adjacent sites to influence C/EBPβ 137 (Fig. 4). Finally, it has also been reported that PPARγ is O‐GlcNAcylated during adipocyte differentiation 126; however, the site of O‐GlcNAcylation has not yet been identified. The function of the O‐GlcNAcylation of the key regulators in adipocyte differentiation should be studied further.

At last, the O‐GlcNAcylation of proteins is global increased in adipogenic differentiation 136, including vimentin, pyruvate carboxylase, ewing sarcoma protein, long‐chain fatty acid‐CoA ligase 1 138 and nucleoporin p62/p98 139, Vimentin 140, pyruvate carboxylase 141 and Ewing sarcoma protein 142 are heavily O‐GlcNAcylated during adipocyte differentiation (Table 2). Further studies should be performed to expand our knowledge of the roles of the O‐GlcNAcylation of these proteins in adipocyte differentiation.

**Table 2:** Proteins that were increasingly O‐GlcNAcylated during 3T3‐L1 pre‐adipocyte differentiation

Protein	MW (kD)	Characteristic	Role	Reference
Vimentin	53.7	Major intermediate filament protein	The arrangement of vimentin intermediate filament changes dynamically from an extended fibrillar state to a complex cage formation tightly associated with the forming lipid droplets during adipocyte differentiation	118
Pyruvate carboxylase	130.3	Enzyme that catalyzes the irreversible carboxylation of pyruvate to form oxaloacetate	Plays a crucial role in gluconeogenesis and lipogenesis, in the biosynthesis of neurotransmitters, and in glucose‐induced insulin secretion by pancreatic islets	119
Ewing sarcoma protein	68.6	A member of the TET (TLS/EWS/TAF15) family of RNA‐ and DNA‐binding proteins whose expression is altered in cancer	Affects transcription and RNA processing and pays a role in homologous recombination, DNA damage response and maintenance of genome integrity	120
Long‐chain fatty acid‐CoA ligase 1	78.9	Isozyme of the long‐chain fatty‐acid‐coenzyme A ligase family	Plays a key role in lipid biosynthesis and fatty acid degradation	116
Nucleoporin p62/p98	53.2/97.9	Proteins which are the constituent building blocks of the nuclear pore complex	Mediates transport of macromolecules between the cell nucleus and cytoplasm in eukaryotes	117

## Conclusions and perspectives

The microenvironment, including glucose level, pH and oxygen level, determines the fate of these cells, and glucose concentration regulates differentiation proficiency. Increasing evidence suggests that O‐GlcNAcylation acts as a nutrient sensor that associates the glucose metabolic status with cellular regulation of signal transduction, transcription, protein function and differentiation. The O‐GlcNAcylation of signalling molecules involved in glucose metabolism and cell differentiation has recently received greater appreciation, and the roles of this modification to signalling molecules in the cytoplasm, nucleus, and mitochondria in regulating cell differentiation with glucose metabolism constitutes an intriguing area of research Because glucose concentrations, protein O‐GlcNAcylation and cell differentiation affect ageing and diseases, uncovering the underlying functions and mechanisms will be very important for exploring glucose or O‐GlcNAcylation as a therapeutic target for diseases.

## Conflicts of interest

The authors confirm that there are no conflicts of interest.
